# Accessible analysis of longitudinal data with linear mixed effects models

**DOI:** 10.1242/dmm.048025

**Published:** 2022-05-06

**Authors:** Jessica I. Murphy, Nicholas E. Weaver, Audrey E. Hendricks

**Affiliations:** 1 Mathematical and Statistical Sciences, University of Colorado Denver, Denver, CO 80217, USA; 2 Biostatistics and Informatics, Colorado School of Public Health, Aurora, CO 80045, USA

**Keywords:** ANOVA, Linear mixed effects, Longitudinal, Microbiome, Mouse, Shiny app

## Abstract

Longitudinal studies are commonly used to examine possible causal factors associated with human health and disease. However, the statistical models, such as two-way ANOVA, often applied in these studies do not appropriately model the experimental design, resulting in biased and imprecise results. Here, we describe the linear mixed effects (LME) model and how to use it for longitudinal studies. We re-analyze a dataset published by Blanton et al. in 2016 that modeled growth trajectories in mice after microbiome implantation from nourished or malnourished children. We compare the fit and stability of different parameterizations of ANOVA and LME models; most models found that the nourished versus malnourished growth trajectories differed significantly. We show through simulation that the results from the two-way ANOVA and LME models are not always consistent. Incorrectly modeling correlated data can result in increased rates of false positives or false negatives, supporting the need to model correlated data correctly. We provide an interactive Shiny App to enable accessible and appropriate analysis of longitudinal data using LME models.

## Abstract (precis)

**Summary:** Using example data in the public domain and a newly developed, accessible web-based analysis tool, linear mixed effects models are demonstrated to be more appropriate for analyzing longitudinal data than ANOVA.

## INTRODUCTION

Longitudinal studies are often used in biomedical research to improve our understanding of human conditions. Common applications for mouse models in particular include studying the effects of the gut microbiome on human health and disease (Blanton et al., 2016; Britton et al., 2019; Feehley et al., 2019; Ridaura et al., 2013; Tanoue et al., 2019), as well as studying working and long-term memory in Alzheimer's disease (Alamed et al., 2006; Cracchiolo et al., 2007; Gajbhiye et al., 2017; Rosenzweig et al., 2019). In these studies, subjects (e.g. mice) are randomly assigned to a treatment group and a continuous outcome, such as growth, is tracked across time. The goal of longitudinal studies is usually to determine whether the trajectories of subjects over time vary by treatment group.

Correlation between observations is inherent within longitudinal studies due to the longitudinal and sometimes crossed or nested study design. For instance, longitudinal measurements taken from the same subject are likely to be more similar to each other than measurements taken from different subjects. Experiments may also have a crossed study design if time is considered categorical instead of continuous and each subject is measured at the same time points. In gut microbiome studies, experiments may have a nested design whereby the fecal sample from a single donor is transplanted into multiple mice (Blanton et al., 2016; Britton et al., 2019; Feehley et al., 2019). Mice with transplanted microbiota from the same donor are likely to have more similar (i.e. correlated) microbiota profiles. Incorrectly modeled experimental structure can result in biased and imprecise estimates, which can lead to inaccurate conclusions (Cheng et al., 2010; Verbeke and Molenberghs, 2000).

Correctly modeling correlated data requires careful consideration. In many longitudinal studies, a two-way ANOVA model is used (Blanton et al., 2016; Ridaura et al., 2013; Tanoue et al., 2019), with predictor variables for treatment group, time, and the interaction between treatment group and time. A two-way ANOVA assumes independent observations and thus does not account for correlation from longitudinal or crossed/nested measurements. Repeated measures ANOVA is also commonly used (Cracchiolo et al., 2007; Rosenzweig et al., 2019), which does control for the correlation of measurements due to longitudinal structure but not for the correlation induced by crossed or nested designs. The linear mixed effects (LME) model is a flexible method enabling correct modeling of both longitudinal and crossed or nested correlation (Feehley et al., 2019).

Here, we describe the LME model and how to use it for longitudinal studies. We also re-analyze a dataset from Blanton et al. published in Science in 2016 that investigated the growth trajectories of mice supplied with fecal microbiota from nourished and malnourished children (Blanton et al., 2016). Finally, we provide an interactive Shiny App so others can easily implement an appropriate statistical analysis for longitudinal studies.

## RESULTS

### Linear mixed effects models

A traditional linear model is defined by the following formula:
(1)

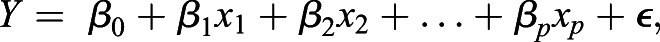
where *Y* is the outcome variable, *x*_1_, …, *x*_*p*_ are the predictor variables, *β*_0_, …, *β*_*p*_ are the regression parameters reflecting the relationship between each *x*_*j*_ and *Y* while controlling for the other predictors, and *ε* is the random error.

When used for longitudinal models, trajectories by group are often modeled using Eqn 2 below:
(2)

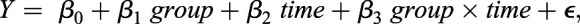
where *group* refers to the treatment group, *time* refers to the time measurement (continuous), and *group×time* refers to the interaction between treatment group and time. The model parameters are defined in Table 1. This equation is identical to a two-factor ANOVA with interaction. The group by time interaction is usually the effect of interest in longitudinal studies and identifies whether trajectories differ by group.

**Table 1.**
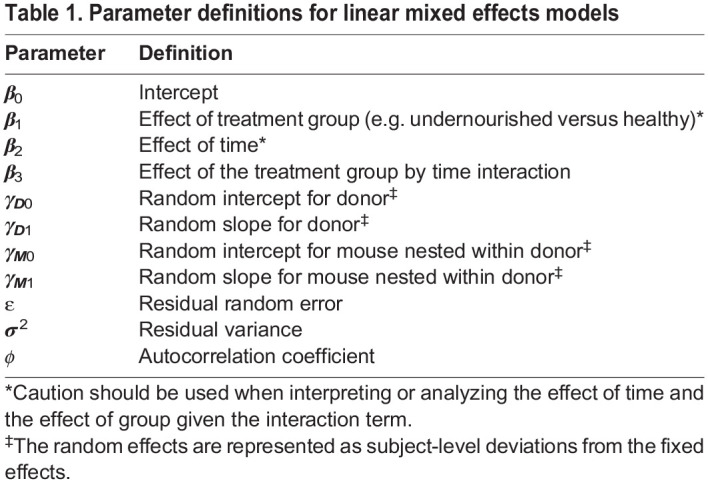
Parameter definitions for linear mixed effects models

The random error term, *ε*, captures variation in the outcome, *Y*, not explained by the model. In a linear model, the random error is assumed to be independent and identically distributed with a mean of zero and a constant variance *σ*^2^ from a normal distribution 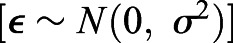. The ANOVA model is reasonably robust to violations in the normality assumption, but not to violations in the assumption of independence. The independence assumption is violated if unaccounted correlation is present, which occurs in longitudinal data and nested designs.

To account for correlation between observations, LMEs use random effects to explicitly model the correlation structure, thus removing correlation from the error term. Random effects can take many functional forms (e.g. linear, quadratic) depending on the structure of the data. Here, we focus on two commonly used random effects: a random intercept and a random slope. For example, a random intercept for subject allows the mean value (i.e. intercept) of *Y* to differ between subjects whereas a random slope term allows the rate of change (i.e. slope) between a predictor, such as time, and the outcome to differ between subjects. A random slope in addition to a random intercept allows both the rate of change and the mean value to vary by subject. Random slopes are usually only used with random intercepts.

A random slope, *γ*_*i*1_, and intercept, *γ*_*i*0_, model for subject *i* is shown in Eqn 3 below:
(3)

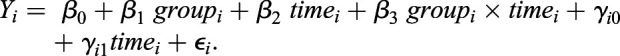
In this model, *β*_0_, *β*_1_, *β*_2_ and *β*_3_ are often referred to as fixed effects because they are the same for all subjects. Random effects are usually specified for the factor (i.e. categorical) variables with multiple levels or categories within the experiment. The random intercept term, *γ*_*i*0_, represents subject-level deviations from the population-level intercepts (e.g. *β*_0_ for the reference group and *β*_0_+*β*_1_ for the treatment group) and the random slope term represents subject-level deviations from the population-level slopes (e.g. *β*_2_ for the reference group and *β*_2_+*β*_3_ for the treatment group). For mouse models with a nested study design, the factor variables are mouse (multiple mice nested within a single donor) and donor (multiple donors per treatment group). The mouse would be considered the nested random effect and the donor would be considered the higher-level random effect (Schielzeth and Nakagawa, 2013).

Autocorrelation between successive time points can be modeled directly through the error term in LMEs. Instead of assuming independent errors, an autoregressive (AR) correlation structure can be specified [*ε*_*t*_ = *φ ε*_*t*−1_ + *Z*_*t*_ with 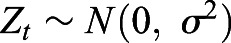 for time point *t* and |*φ*| < 1]. This structure assumes that observations closer together in time are more related than observations farther apart, which is often true in longitudinal studies. Whereas random effects are used to model the variation between different subjects, correlation structures for the errors are used to model the variation within subjects over an ordered structure, such as time.

After specifying a preliminary mean structure (Eqn 2), a preliminary random effects structure (Eqn 3) and a residual covariance structure, model reduction can be performed if appropriate (Verbeke and Molenberghs, 2000). When in doubt or if the structure of the data is unknown, the maximal model, which includes a slope and intercept for each random effect consistent with the study design, is commonly used (Barr et al., 2013; Schielzeth and Forstmeier, 2009; Verbeke and Molenberghs, 2000; Cheng et al., 2010). Then, the need for each slope and/or intercept can be tested for inclusion in the model as further described (Matuschek et al., 2017).

### Re-analysis of the Blanton et al. dataset

Eight models were evaluated in the re-analysis of the Blanton et al. dataset (Blanton et al., 2016). The models are described in the following section, and the model parameters are defined in Table 1. The maximal model (Model I) is specified below, where *Y* refers to the outcome variable (growth), *group* refers to the treatment group (undernourished versus healthy), *time* refers to the number of days, and *group*×*time* refers to the interaction between treatment group and time for subject *i*. The nested models (Models II-V) include a subset of terms from the maximal model. The autocorrelation models (Models VI-VIII) include error terms with autoregressive correlation structures whereas the maximal and nested models include independent errors.

#### Maximal model 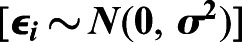


Donor intercept and slope plus mouse intercept and slope random effects:
(rmModelI)

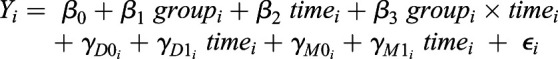


#### Nested models 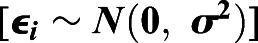


Donor intercept plus mouse intercept and slope random effects:
(rmModelII)

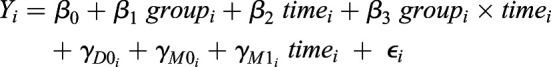
Mouse intercept and slope random effects:
(rmModelIII)

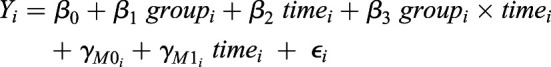
Mouse intercept random effect:
(rmModelIV)

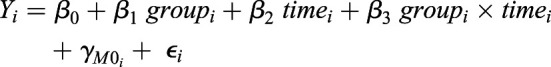
No random effects:
(rmModelV)

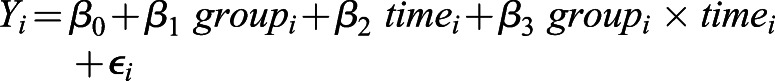


#### Autocorrelation models [ε_*i*,*t*_ = *φ*ε_*i*,*t*−1_ + *Z*_*i*,*t*_ with 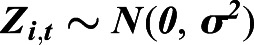 for time point *t* and |*φ*| < 1]

Mouse slope random effect:
(rmModelVI)

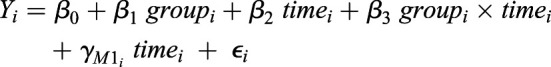
Mouse intercept random effect:
(rmModelVII)

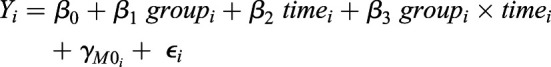
No random effects:
(rmModelVIII)

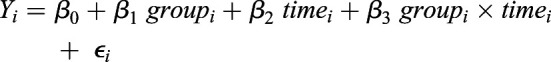


To test if the study design used by Blanton et al. resulted in correlation between observations within donor and mouse, hypothesis tests of the random effect terms (i.e. tests of nested models) were performed with a likelihood ratio test (LRT) as well as a parametric bootstrap test. For both, each additional random effect term significantly improved model fit, indicating that the maximal model (Model I) is preferred out of the nested models (Table 2). However, Model I produced convergence warnings for three out of seven optimizers checked (bobyqa, Nelder_Mead and optimx.L-BFGS-B), but the parameter estimates were consistent among the optimizers that converged. Model II was significantly better than Model III and did not produce any convergence warnings. Residual profile plots of the nested models (Fig. S1) showed that Model V had the worst fit followed by Model IV, with Models I-III having the best fit.

**Table 2.**
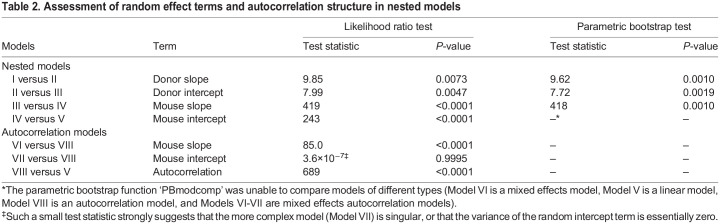
Assessment of random effect terms and autocorrelation structure in nested models

Model fit improved when an autocorrelation structure was used for the residual error (Model VIII versus V) and a random slope for mouse was added (Model VI versus VIII). However, the addition of a random intercept for mouse was unnecessary when accounting for autocorrelation (Model VII versus VIII) given that the estimated variance of the random intercept term was very close to zero. Therefore, Model VI only contains a random slope for mouse without a random intercept, which is still consistent with the study design as growth was measured as change from baseline, thus forcing the intercept to be zero for all mice.

The interaction effect estimate is smaller for the linear model (Model V) and the autocorrelation models without a random slope (Models VII and VIII) compared to the other models (Models I-IV and VI) for which the estimates are fairly consistent. The standard error estimates for the interaction effect vary more between models. The standard error estimate of the interaction term is largest for the maximal model (Model I) and smallest for the simpler models (Models IV-V and VII-VIII) (Table 3).

**Table 3.**
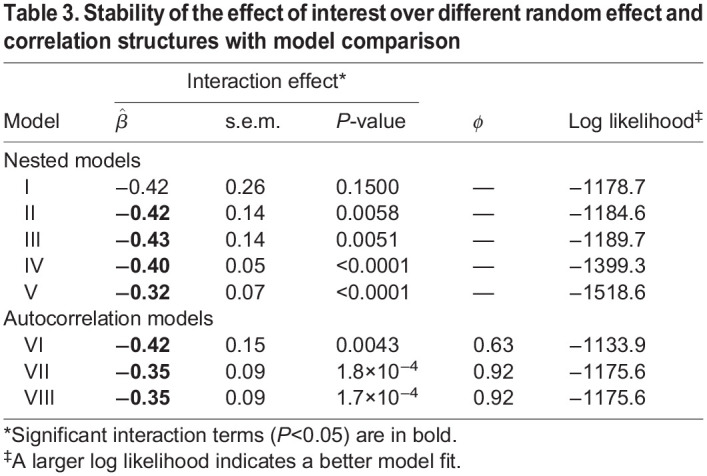
Stability of the effect of interest over different random effect and correlation structures with model comparison

The autocorrelation models fit the data better than the nested models, with the simpler autocorrelation models (Models VII and VIII) having similar log likelihoods to the maximal model (Model I). Model VI, the autocorrelation model with a random slope for mouse, had the best fit overall with the largest log likelihood (Table 3). This ‘best’ model was chosen based on the study design and model fit, not the *P*-value of the effect of interest.

### Simulations

To compare the type I error and power of the models, data were simulated for three different covariance scenarios: a random intercept and slope for mouse (Model III), a random intercept for mouse (Model IV), and no random effects (Model V). Across all simulation scenarios, the model that matches the simulation scenario performs best by maintaining the type I error and having higher power (Table 4 and Fig. S2). For instance, for the ‘mouse intercept and slope’ simulation scenario, the model that matches the simulation scenario (Model III) is the only model that maintains the appropriate type I error. The type I error is severely inflated for Models IV and V, which do not include the slope random effect term. For the ‘mouse intercept’ simulation scenario, Model IV, which matches the simulation scenario, has slightly more power than Models III and V. But, the type I error is very conservative for all three models, even the model that matches the simulation scenario (Model IV). Table S1 highlights the increased power for Model IV more clearly as the type I error is no longer conservative for this larger sample size (*n*=40 independent mice). For the simulation scenario with no random effects, Model V, which matches the simulation scenario, and Model IV are both close to the expected value of 0.05, whereas Model III has a slightly conservative type I error estimate. Consequently, Model III also has slightly less power than Models IV and V.

**Table 4.**
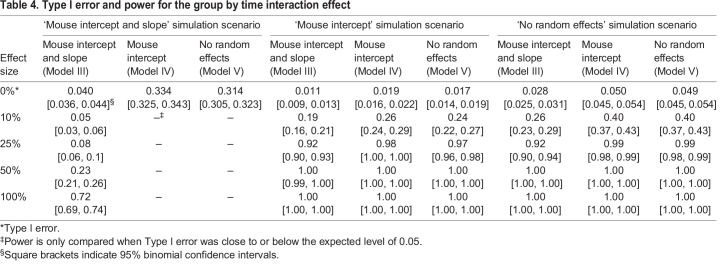
Type I error and power for the group by time interaction effect

To determine whether the small simulated sample size of eight donors with five mice nested within each donor was affecting the type I error estimates, a simulation analysis was performed for larger sample sizes (Table 5). The nested model of five mice per eight donors results in 40 mice, but mice within the same donor are correlated (i.e. contain dependent information), resulting in an effective independent sample size less than 40. Conversely, the scenario with 40 donors, one for each mouse, provides independent information for each mouse. Indeed, for the ‘mouse intercept’ simulation scenario, the type I error for Model IV is deflated for the five mice per eight donors sample size, but is close to the expected value of 0.05 when there are 40 independent mice. The over-conservative type I error could be due to a failure in approximation for the Wald test at small sample sizes (Verbeke and Molenberghs, 2000). For the ‘mouse intercept’ simulation scenario, Models III and V, which do not match the experimental design of the simulation scenario, have slightly and considerably deflated type I error, respectively. Model III, which contains an unnecessary random slope term, approaches the expected type I error as the sample size increases. Model V, which does not include the necessary random intercept term, has severely deflated type I error across all sample sizes. For the ‘mouse intercept and slope’ simulation scenario, the type I errors for Models IV and V are severely inflated across all sample sizes.

**Table 5.**
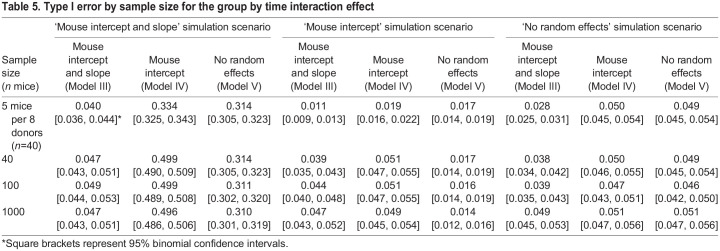
Type I error by sample size for the group by time interaction effect

These results suggest that leaving out a necessary random effect term will result in an inaccurate type I error even at large sample sizes. This is seen with Models IV and V for the ‘mouse intercept and slope’ simulation scenario and with Model V for the ‘mouse intercept’ simulation scenario. Importantly, the direction of the bias in the type I error can result in too conservative (i.e. too small type I error) or anti-conservative (i.e. too large type I error) results. Models that include an unnecessary random effect term, e.g. Model III in the ‘mouse intercept’ simulation scenario or Models III or IV in the ‘no random effects’ simulation scenario, can result in deflated type I error at smaller sample sizes. However, this bias decreases as the sample size increases, ultimately resulting in the appropriate type I error. These results suggest that the type I error is better controlled when random effect terms are included, but may result in lower power, as shown in Table 2. Regardless, as we discuss above and show in Table 2, whether including random effect terms results in a better model fit can be tested while defaulting to the maximal model consistent with the study design (Schielzeth and Forstmeier, 2009).

### EasyLME: a Shiny app

**Fig. 1.**
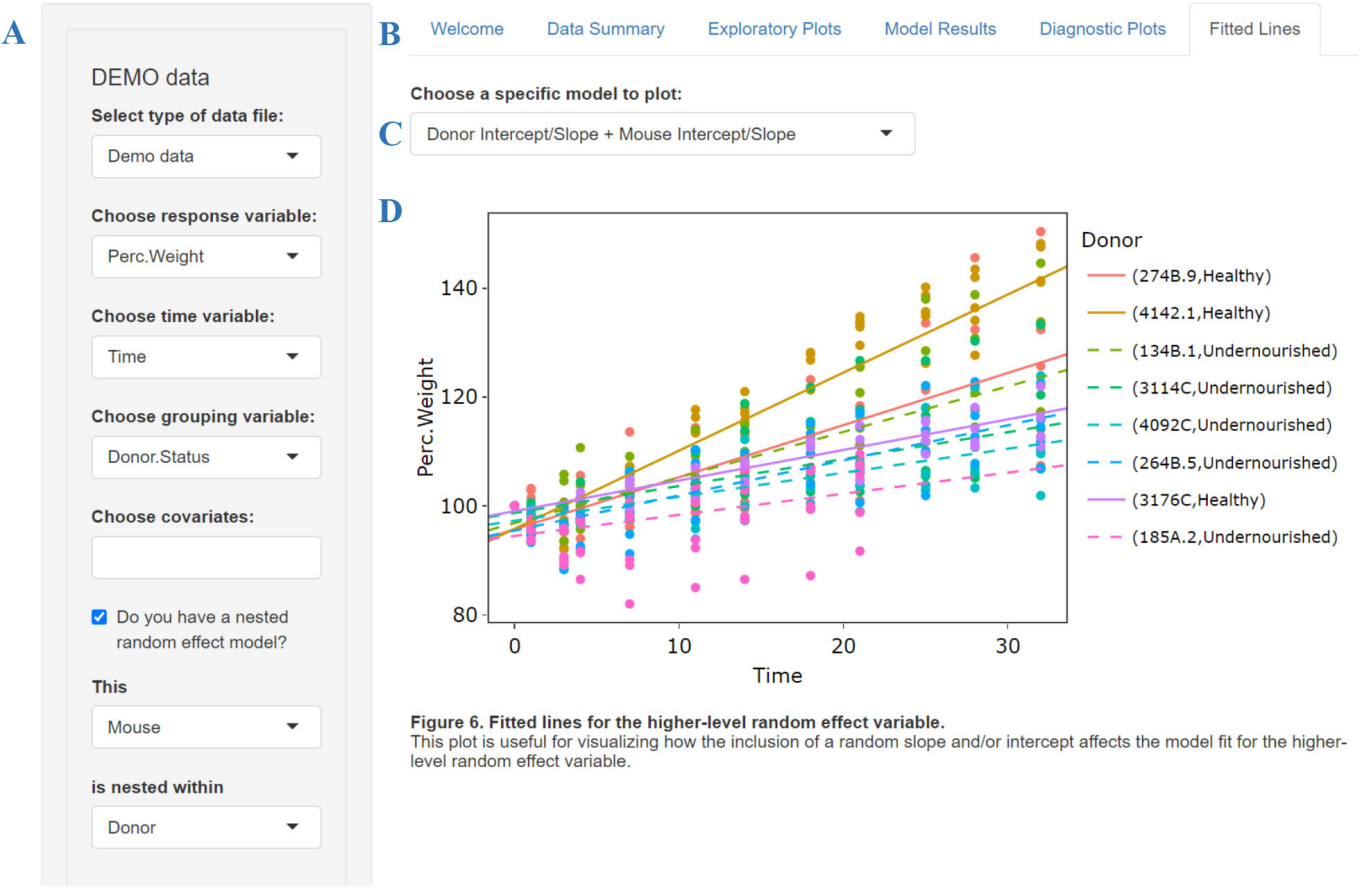
**EasyLME Shiny app user interface featuring the ‘Fitted Lines’ tab.** (A) Default variables for the Blanton et al. demo data. (B) Main tabs of the app. (C) Drop-down menu to select a specific model to visualize the fitted lines. (D) Plot of the original data and fitted lines for the higher-level random effect variable in the maximal model (Donor Intercept/Slope+Mouse Intercept/Slope).

Because analyzing longitudinal data requires careful consideration and the use of LME models is not always easily available, we provide an interactive and user-friendly Shiny app (https://cran.r-project.org/package=shiny) that allows others to implement and choose an appropriate LME model more easily. The app, called EasyLME, can be accessed through any web browser at https://shiny.clas.ucdenver.edu/EasyLME/. To explore the app's features, users can upload their own data or use the demo data from Blanton et al. Users can then select variables representing the structure of their data, including an option to specify whether the model contains nested random effects. Users can also select additional covariates if desired. The app assumes that the response and time variables are continuous and the grouping variable as well as the random effects are factors. However, users should code categorical covariates (e.g. sex) as characters (e.g. male/female); otherwise, numerically coded covariates will be treated as continuous. For the demo data, the random effect variables are ‘Mouse’ nested in ‘Donor’, as shown on the left-hand side of Fig. 1, because samples from each donor were transplanted into multiple mice. Once the variables have been chosen, the user can navigate through the output tabs to see the various aspects of the analysis. This basic design of the app was inspired by medplot, a Shiny app for longitudinal medical data (Ahlin et al., 2015).

The app contains six tabs, as shown in Fig. 1: ‘Welcome’, ‘Data Summary’, ‘Exploratory Plots’, ‘Model Results’, ‘Diagnostic Plots’ and ‘Fitted Lines’. The main page of the app is the ‘Welcome’ tab with an ‘About’ section explaining the app functions followed by a ‘Getting Started’ section. The ‘Data Summary’ tab provides univariate summaries of each variable selected by the user grouped by the random effect variable (or by the higher-level random effect if the data are nested). The data summaries help to ensure that the appropriate variables are selected and can highlight any extreme or missing values.

**Fig. 2.**
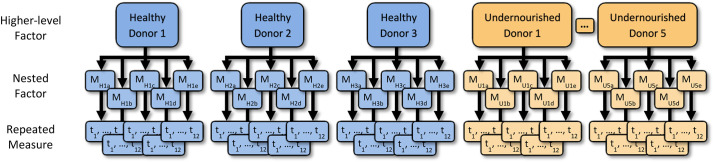
**Study design for the Blanton et al. dataset.** Fecal samples from children aged 6-18 months were orally transferred to five germ-free mice (M). The analysis was restricted to the three healthy (H) and five undernourished (U) donor samples that produced >50% transplantation efficiency. The percentage weight change of each mouse was recorded at 12 time points (t_1-12_): 0, 1, 3, 4, 7, 11, 14, 18, 21, 25, 28 and 32 days.

The ‘Exploratory Plots’ tab provides three visualizations helpful to understand structure in the data. The scatterplot in figure 1 of the app (Fig. S3) shows the relationship between the response variable and time. This plot is useful for checking the assumption of linearity between the response and time. The trendlines in figure 2 of the app (Fig. S4) display the average response over time for the higher-level factor (e.g. donor). This plot helps to visualize whether a random intercept and/or random slope would be appropriate for the higher-level random effect. It also shows the presence of missing data if there are gaps in the lines or the lines stop short. If a user's data are not nested, this graph will just show the trendlines for the random effect, without the need for averaging. The third figure in the ‘Exploratory Plots’ tab (Fig. S5) shows the trendlines for the nested random effect (e.g. mouse) faceted by the higher-level random effect (e.g. donor). This plot is helpful to visualize whether a random intercept and/or random slope would be appropriate for the nested random effect. If a user's data is not nested, this plot will not be displayed.

The ‘Model Results’ tab contains a comparison table for testing nested models. The table contains coefficient estimates with standard errors in parentheses, log likelihoods, and *P*-values from likelihood ratio tests of the nested models. These results are similar to those shown in Tables 2 and 3. If a user's data are nested (e.g. multiple mice per donor), the table compares the five nested models listed above in the ‘Re-analysis of Blanton et al. dataset’ section. If the data are not nested (e.g. one mouse per donor), the table only compares Models III-V (mouse intercept and slope, mouse intercept, and no random effects). All models are assumed to have a simple residual covariance structure 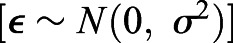; more complex structures, such as autocorrelation, will be supported in the next version of the app. A coefficient plot (Fig. S6) is also provided below the table to allow a visual comparison of the 95% confidence intervals of the fixed effect estimates for the different models. Below the plot, the user can select a specific LME model from the drop-down menu to see more detailed information, such as the random effect estimates.

Convergence warnings, if any, for seven different optimizers are provided in the ‘Model Results’ tab. For singular fit warnings, we recommend the user choose a simpler model that excludes random effect terms estimated to be at or very close to zero. The user could also center and scale the continuous predictor variables to possibly resolve convergence warnings. Other potential fixes, such as increasing the tolerance level of the optimizer or using different starting values for the parameters, are currently not provided in the app but could be implemented directly within the lmer function (Bates et al., 2015). In the next version of the app, the user will have the ability to change the optimizer and refit the selected model if needed.

The subsequent ‘Diagnostic Plots’ tab provides visualizations to compare model fits. Figure 4 of the app (Fig. S1) shows residual profile plots in increasing order of model complexity. Models with a large, nonconstant variability in the residuals over time indicate a worse model fit, whereas models with a small, constant variability in the residuals over time indicate a better model fit.

Lastly, given a selected model, the ‘Fitted Lines’ tab visualizes the fitted line for each random effect factor (e.g. donor or mouse) as seen in Fig. 1. If a user's data are nested and the selected model contains both random effects, then two plots will be displayed, one for each of the nested and higher-level random effects (e.g. mouse and donor). These plots are useful for visualizing how the inclusion of a random slope and intercept versus just a random intercept affects the model fit. The inclusion of a random intercept versus no random effects can also be compared. If a user's data are not nested or the selected model just contains one random effect, then only one plot will be displayed for the specific random effect variable.

All plots within the app are made with plotly (Sievert, 2020) and are thus interactive and can be downloaded as a PNG. Specifically, users can zoom the plots in and out, hover over data points to display more information, and click on the legend entries to remove a group of points from the plot (clicking again will add the points back).

## DISCUSSION

Longitudinal data, and especially nested longitudinal data, are often modeled incorrectly. ANOVA, although commonly used, does not accurately account for correlation from repeated measurements or nested structure. Here, we compare the LME model with ANOVA in a gut microbiome case study involving longitudinal mouse models. In our re-analysis of data published by Blanton et al. (2016), we find evidence that LME models based on the experimental design better fit the data. Importantly, we show that results from the LME model and ANOVA will not always produce consistent results in terms of effect estimate, standard error and, subsequently, statistical significance. Through simulations, we show that the type I error is not well controlled when using an ANOVA model for longitudinal data and can result in false positives or false negatives. We also show that LMEs, with the inclusion of necessary random effect terms to model the experimental structure appropriately, produce unbiased type I error at sufficiently large sample sizes.

To allow easier implementation of LME models, we designed EasyLME, a Shiny app to enable appropriate analysis of longitudinal studies. The app's easy-to-use features, such as the exploratory plots, model tests/comparisons, and fitted lines, allow researchers to choose the most suitable model for their data. Although the demo data pertains to mouse models, the app is applicable to longitudinal studies in general to identify group differences in trajectory for a continuous outcome. For a more comprehensive open-source tool, we recommend jamovi (https://www.jamovi.org/), which also includes LMEs in the GAMLj module (https://blog.jamovi.org/2018/11/13/introducing-gamlj.html).

Although the goal of the app is to make analyzing longitudinal data with LMEs more accessible, consultation with a statistician is still recommended in study design, analysis and interpretation of results. We also recommend that, prior to analysis, users create a data analysis plan that describes quality control, significance thresholds and how the final model will be chosen (Cheng et al., 2010; Michener, 2015; Simpson, 2015). Additionally, we advise the user to consider both model fit and stability when selecting a model (e.g. visualizations, nested model comparisons, as well as convergence warnings). If the results and conclusions vary greatly between the models, such as large changes in the effect size or significance of the interaction term, we recommend reporting the results from all models considered. Additional structure could still exist within the data, such as autocorrelation, which would require further investigation. Generalized additive models could potentially be used to explain nonlinear patterns in the data given enough time points (McEvoy-May et al., 2021).

Although LME models can be performed in other programs (e.g. Stata, SAS, SPSS), R was used for this analysis because it is a widely known, free and open-source platform. As such, we hope our app will increase accessibility for applied researchers working with longitudinal data.

## MATERIALS AND METHODS

### Blanton et al. dataset

The dataset used in this paper was obtained from a 2016 publication entitled ‘Gut bacteria that prevent growth impairments transmitted by microbiota from malnourished children’ (Blanton et al., 2016). In the experiment of interest, fecal samples from eight healthy and eleven undernourished children aged 6 or 18 months were obtained. Each fecal sample was orally transferred to five germ-free mice. The percentage weight change of each mouse was recorded at 12 time points: 0, 1, 3, 4, 7, 11, 14, 18, 21, 25, 28 and 32 days. In the original study, the statistical analysis was restricted to eight donor samples (three healthy, five underweight) that produced >50% transplantation efficiency resulting in a total of 480 observations across all time points, mice and donors. Forty-three observations were missing, resulting in 437 observations. The study design for this dataset is shown in Fig. 2.

### Re-analysis of Blanton et al. dataset

Analysis of the Blanton et al. data with LME models was performed using the nlme v3.1-149, lme4 v1.1-23 and lmerTest v3.1-2 packages in R v4.0.3 (Bates et al., 2015; Kuznetsova et al., 2017; R Core Team, 2020; https://cran.r-project.org/package=nlme). The coefficients were estimated using restricted maximum likelihood (REML) (Verbeke and Molenberghs, 2000) and the random effects terms were allowed to be correlated. The fit of each model was measured using log likelihoods, with a larger log likelihood indicating a better fit. The stability of the model estimates for different optimizers was checked using the allFit function from the lme4 v1.1-23 R package (Bates et al., 2015). The estimates were considered stable if they did not change substantially across the optimizers.

The LRT is an appropriate method for hypothesis testing of fixed effects terms. However, the LRT can produce inaccurate *P*-values for random effects because random effect estimates are tested against zero, which is on the boundary of the parameter space (variance must be greater than or equal to zero) (Verbeke and Molenberghs, 2000). As such, parametric bootstrap methods (PBmodcomp function from the pbkrtest package) in addition to LRTs were used to perform hypothesis tests of random effect terms (Halekoh and Højsgaard, 2014). The PBmodcomp function can only be used to compare models of the same type and thus could not be used to test an LME model (Model IV) versus a linear model (Model V), an autocorrelation model (Model VIII) versus a linear model (Model V), or a mixed effects autocorrelation model (Models VI-VII) versus an autocorrelation model (Model VIII). When completing hypothesis tests of nested models, each model was refit using maximum likelihood (ML) estimation. For all statistical tests, a significance threshold of *P*<0.05 was used.

Model fit was visualized with residual profile plots. Residuals represent the difference between the observed response value and the estimated response value from the model. Models that do not fit the data well produce estimates farther from the truth (i.e. larger residuals). Patterns or nonconstant variability in the residuals over time can also indicate a poor model fit. In the profile plots, where each line represents a single profile (i.e. mouse), the residual trendlines of the best fitting models form a small, constant band around zero.

### Simulations

Data were simulated from a multivariate normal distribution (mvrnorm function from the MASS package) for three different covariance scenarios: a random intercept and slope for mouse (Model III), a random intercept for mouse (Model IV), and no random effects (Model V) (Venables and Ripley, 2002). The simulation sample size consisted of four healthy donors and four undernourished donors, with five mice per donor. The same twelve time points used in Blanton et al. were used for simulations: 0, 1, 3, 4, 7, 11, 14, 18, 21, 25, 28 and 32 days. The simulation parameters for the fixed effect estimates for treatment group, time, and time by treatment interaction from Model III were used for all simulation scenarios because this model produced the largest interaction effect. The interaction effect between treatment group and time was varied to be 0, 10, 25, 50 and 100% of the observed effect from Model III for the Blanton et al. data, while keeping the simulation parameters for the group and time effect estimates the same. The mice were assumed to be independent and the same variance matrix was assumed for all mice within a simulation scenario.

LME models III, IV and V were fit to the simulated data using the default optimizer, NLOPT_LN_BOBYQA, within lme4. Type I error and power were calculated using 10,000 and 1000 simulation replicates, respectively. Type I error was assessed for an interaction effect of 0. Because the asymptotic properties of LME models may not be reached for small sample sizes, type I error was also evaluated at larger sample sizes (*n*=40, 100, 1000), with one mouse per donor and an equal number of donors per treatment group. All hypothesis tests were assessed at α=0.05.

## Supplementary Material

Supplementary information

## References

[DMM048025C1] Ahlin, Č., Stupica, D., Strle, F. and Lusa, L. (2015). medplot: a web application for dynamic summary and analysis of longitudinal medical data based on R. *PLoS One* 10, e0121760. 10.1371/journal.pone.0121760 25837352 PMC4383594

[DMM048025C2] Alamed, J., Wilcock, D. M., Diamond, D. M., Gordon, M. N. and Morgan, D. (2006). Two-day radial-arm water maze learning and memory task; robust resolution of amyloid-related memory deficits in transgenic mice. *Nat. Protoc.* 1, 1671. 10.1038/nprot.2006.275 17487150

[DMM048025C3] Barr, D. J., Levy, R., Scheepers, C. and Tily, H. J. (2013). Random effects structure for confirmatory hypothesis testing: Keep it maximal. *J. Mem. Lang.* 68, 255-278. 10.1016/j.jml.2012.11.001 PMC3881361 24403724

[DMM048025C4] Bates, D., Mächler, M., Bolker, B. and Walker, S. (2015). Fitting linear mixed-effects models using lme4. *J. Stat. Softw.* 67, 1-48. 10.18637/jss.v067.i01

[DMM048025C5] Blanton, L. V., Charbonneau, M. R., Salih, T., Barratt, M. J., Venkatesh, S., Ilkaveya, O., Subramanian, S., Manary, M. J., Trehan, I., Jorgensen, J. M. et al. (2016). Gut bacteria that prevent growth impairments transmitted by microbiota from malnourished children. *Science* 351, aad3311. 10.1126/science.aad3311 26912898 PMC4787260

[DMM048025C6] Britton, G. J., Contijoch, E. J., Mogno, I., Vennaro, O. H., Llewellyn, S. R., Ng, R., Li, Z., Mortha, A., Merad, M., Das, A. et al. (2019). Microbiotas from humans with inflammatory bowel disease alter the balance of gut Th17 and RORγt+ regulatory T cells and exacerbate colitis in mice. *Immunity* 50, 212-224.e4. 10.1016/j.immuni.2018.12.015 30650377 PMC6512335

[DMM048025C8] Cheng, J., Edwards, L. J., Maldonado-Molina, M. M., Komro, K. A. and Muller, K. E. (2010). Real longitudinal data analysis for real people: building a good enough mixed model. *Stat. Med.* 29, 504-520. 10.1002/sim.3775 20013937 PMC2811235

[DMM048025C9] Cracchiolo, J. R., Mori, T., Nazian, S. J., Tan, J., Potter, H. and Arendash, G. W. (2007). Enhanced cognitive activity—over and above social or physical activity-is required to protect Alzheimer's mice against cognitive impairment, reduce Aβ deposition, and increase synaptic immunoreactivity. *Neurobiol. Learn. Mem.* 88, 277-294. 10.1016/j.nlm.2007.07.007 17714960 PMC2083653

[DMM048025C10] Feehley, T., Plunkett, C. H., Bao, R., Hong, S. M. C., Culleen, E., Belda-Ferre, P., Campbell, E., Aitoro, R., Nocerino, R. and Paparo, L. (2019). Healthy infants harbor intestinal bacteria that protect against food allergy. *Nat. Med.* 25, 448. 10.1038/s41591-018-0324-z 30643289 PMC6408964

[DMM048025C11] Gajbhiye, K. R., Gajbhiye, V., Siddiqui, I. A., Pilla, S. and Soni, V. (2017). Ascorbic acid tethered polymeric nanoparticles enable efficient brain delivery of galantamine: An in vitro-in vivo study. *Sci. Rep.* 7, 11086. 10.1038/s41598-017-11611-4 28894228 PMC5594022

[DMM048025C12] Halekoh, U. and Højsgaard, S. (2014). A Kenward-Roger approximation and parametric bootstrap methods for tests in linear mixed models - the R package pbkrtest. *J. Stat. Softw.* 59, 1-30. 10.18637/jss.v059.i0926917999

[DMM048025C13] Kuznetsova, A., Brockhoff, P. B. and Christensen, R. H. (2017). lmerTest package: tests in linear mixed effects models. *J. Stat. Softw.* 82, 1-26. 10.18637/jss.v082.i13

[DMM048025C14] Matuschek, H., Kliegl, R., Vasishth, S., Baayen, H. and Bates, D. (2017). Balancing Type I error and power in linear mixed models. *J. Mem. Lang.* 94, 305-315. 10.1016/j.jml.2017.01.001

[DMM048025C15] McEvoy-May, J. H., Jones, D. E., Stoa, L., Dixon, D.-L., Tai, T., Hooker, A. M., Boreham, D. R. and Wilson, J. Y. (2021). Unchanged cardiovascular and respiratory outcomes in healthy C57Bl/6 mice after in utero exposure to ionizing radiation. *Int. J. Radiat. Biol.* 97, 131-138. 10.1080/09553002.2021.1855372 33258723

[DMM048025C16] Michener, W. K. (2015). Ten simple rules for creating a good data management plan. *PLoS Comput. Biol.* 11, e1004525. 10.1371/journal.pcbi.1004525 26492633 PMC4619636

[DMM048025C18] R Core Team. (2020). *R: A Language and Environment for Statistical Computing*. Vienna, Austria: R Foundation for Statistical Computing.

[DMM048025C19] Ridaura, V. K., Faith, J. J., Rey, F. E., Cheng, J., Duncan, A. E., Kau, A. L., Griffin, N. W., Lombard, V., Henrissat, B. and Bain, J. R. (2013). Cultured gut microbiota from twins discordant for obesity modulate adiposity and metabolic phenotypes in mice. *Science (New York, NY)* 341, 1241214. 10.1126/science.1241214 PMC3829625 24009397

[DMM048025C20] Rosenzweig, N., Dvir-Szternfeld, R., Tsitsou-Kampeli, A., Keren-Shaul, H., Ben-Yehuda, H., Weill-Raynal, P., Cahalon, L., Kertser, A., Baruch, K. and Amit, I. (2019). PD-1/PD-L1 checkpoint blockade harnesses monocyte-derived macrophages to combat cognitive impairment in a tauopathy mouse model. *Nat. Commun.* 10, 465. 10.1038/s41467-019-08352-5 30692527 PMC6349941

[DMM048025C21] Schielzeth, H. and Forstmeier, W. (2009). Conclusions beyond support: overconfident estimates in mixed models. *Behav. Ecol.* 20, 416-420. 10.1093/beheco/arn145 19461866 PMC2657178

[DMM048025C22] Schielzeth, H. and Nakagawa, S. (2013). Nested by design: model fitting and interpretation in a mixed model era. *Method. Ecol. Evol.* 4, 14-24. 10.1111/j.2041-210x.2012.00251.x24455157

[DMM048025C23] Sievert, C. (2020). *Interactive Web-Based Data Visualization with R, Plotly, and Shiny*: Chapman and Hall/CRC.

[DMM048025C24] Simpson, S. H. (2015). Creating a data analysis plan: What to consider when choosing statistics for a study. *Can. J. Hosp. Pharm.* 68, 311. 10.4212/cjhp.v68i4.1471 26327705 PMC4552232

[DMM048025C25] Tanoue, T., Morita, S., Plichta, D. R., Skelly, A. N., Suda, W., Sugiura, Y., Narushima, S., Vlamakis, H., Motoo, I. and Sugita, K. (2019). A defined commensal consortium elicits CD8 T cells and anti-cancer immunity. *Nature* 565, 600. 10.1038/s41586-019-0878-z 30675064

[DMM048025C26] Venables, W. N. and Ripley, B. D. (2002). *Modern Applied Statistics with S*. New York: Springer.

[DMM048025C27] Verbeke, G. and Molenberghs, G. (2000). *Linear Mixed Models for Longitudinal Data*. New York, NY: Springer New York.

