# Supplementary material for: Accessible analysis of longitudinal data with linear mixed effects models
Source: Dis Model Mech. 2022 May 6;15(5):dmm048025. doi: 10.1242/dmm.048025 (PMC9092652; doi:10.1242/dmm.048025)
Supplement: Supplementary information [file dmm-15-048025-s1.pdf]

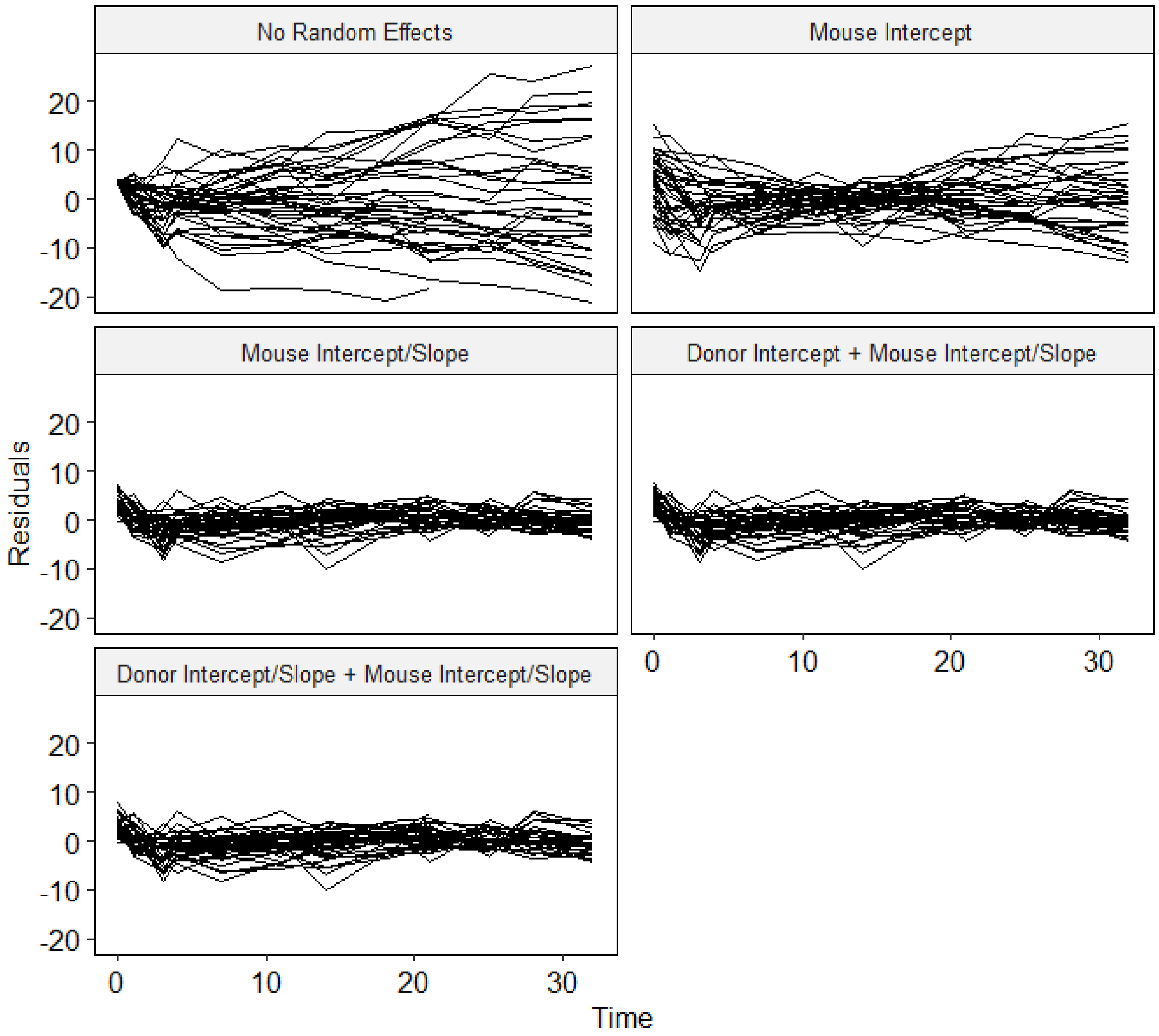

Fig. S1. EasyLME Diagnostic Plots Figure 5. Residual profile plots of model residuals vs time in increasing order of model complexity. Each line represents a single profile of the random effect variable (here Mouse). These plots are useful to visually compare the different model fits. Models with a large, nonconstant variability in the residuals over time (e.g. the No Random Effects model) indicate a worse model fit, whereas models with a small, constant variability in the residuals over time (e.g. the Mouse Intercept/Slope, Donor Intercept + Mouse Intercept/Slope, and Donor Intercept/Slope + Mouse Intercept/Slope models) indicate a better model fit.

Disease Models & Mechanisms • Supplementary information

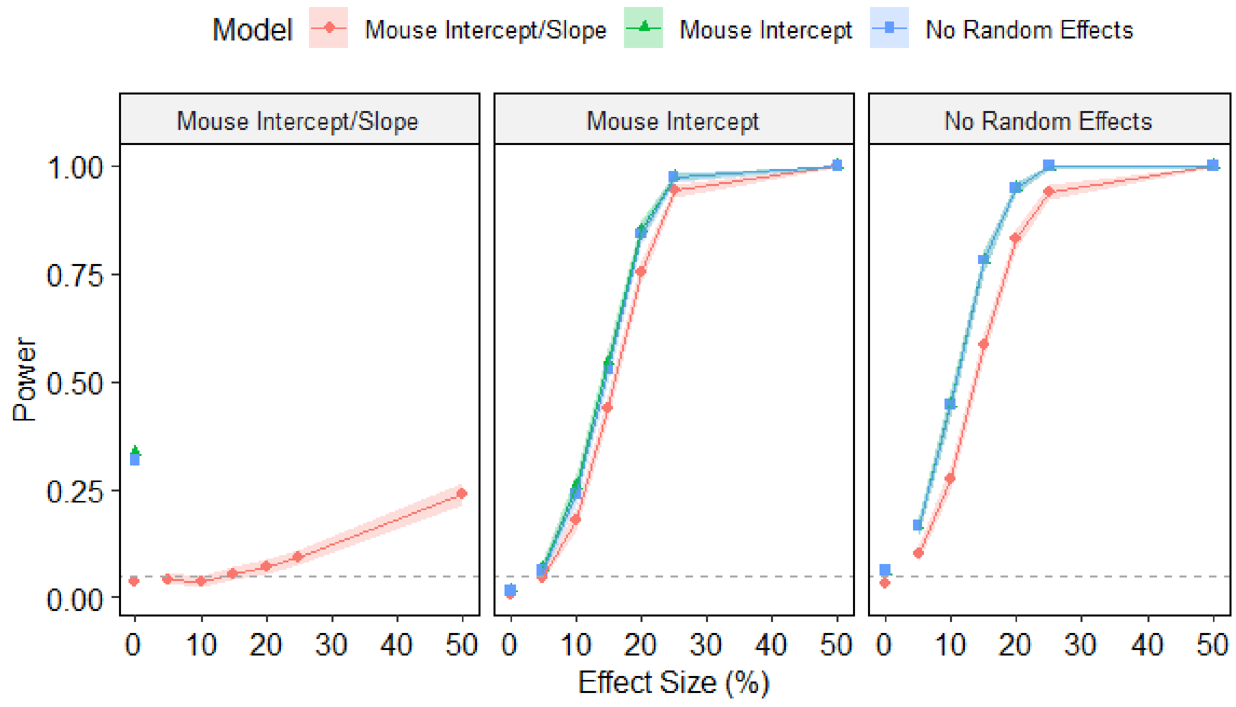

Fig. S2. Type I error and power for the group by time interaction effect. Graphical representation of Table 4 in the main text for three different simulation scenarios: a random intercept and slope for mouse (Mouse Intercept/Slope), a random intercept for mouse (Mouse Intercept), and no random effects (No Random Effects). The interaction effect between treatment group and time was varied to be 0, 5, 10, 15, 20, 25, and 50% of the observed effect from the Mouse Intercept/Slope model for the Blanton et al. data. The Mouse Intercept/Slope, Mouse Intercept, and No Random Effects models were fit to the simulated data. Type I error and power were calculated using 1,000 simulation replicates. Type I error was assessed for an interaction effect of 0 shown here as single points. Power is shown as points connected by a line with a 95% shaded confidence band. All hypothesis tests were assessed at $\alpha = 0.05$ shown here as a horizontal dashed line.

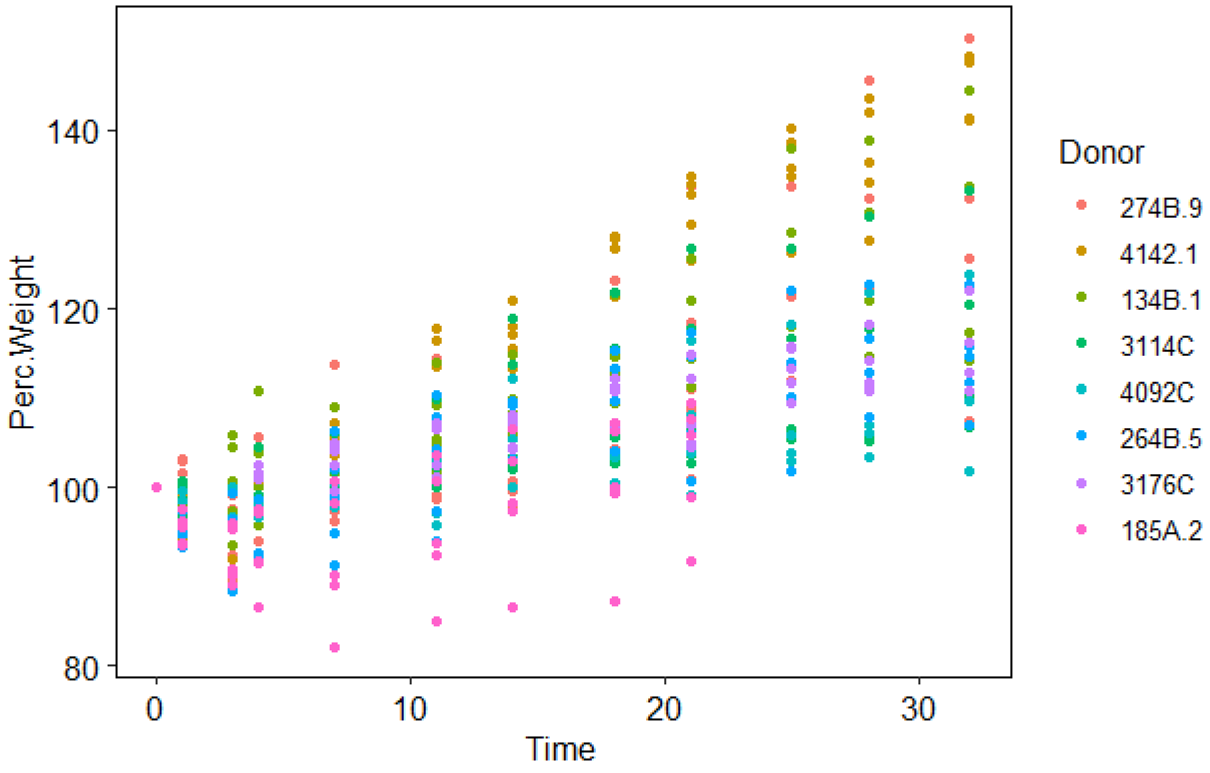

Fig. S3. EasyLME Exploratory Plots Figure 1. Scatterplot of the response variable (here Perc.Weight) vs time with observations colored by the higher-level random effect variable (here Donor). This plot useful for checking the linearity assumption between the response variable and time.

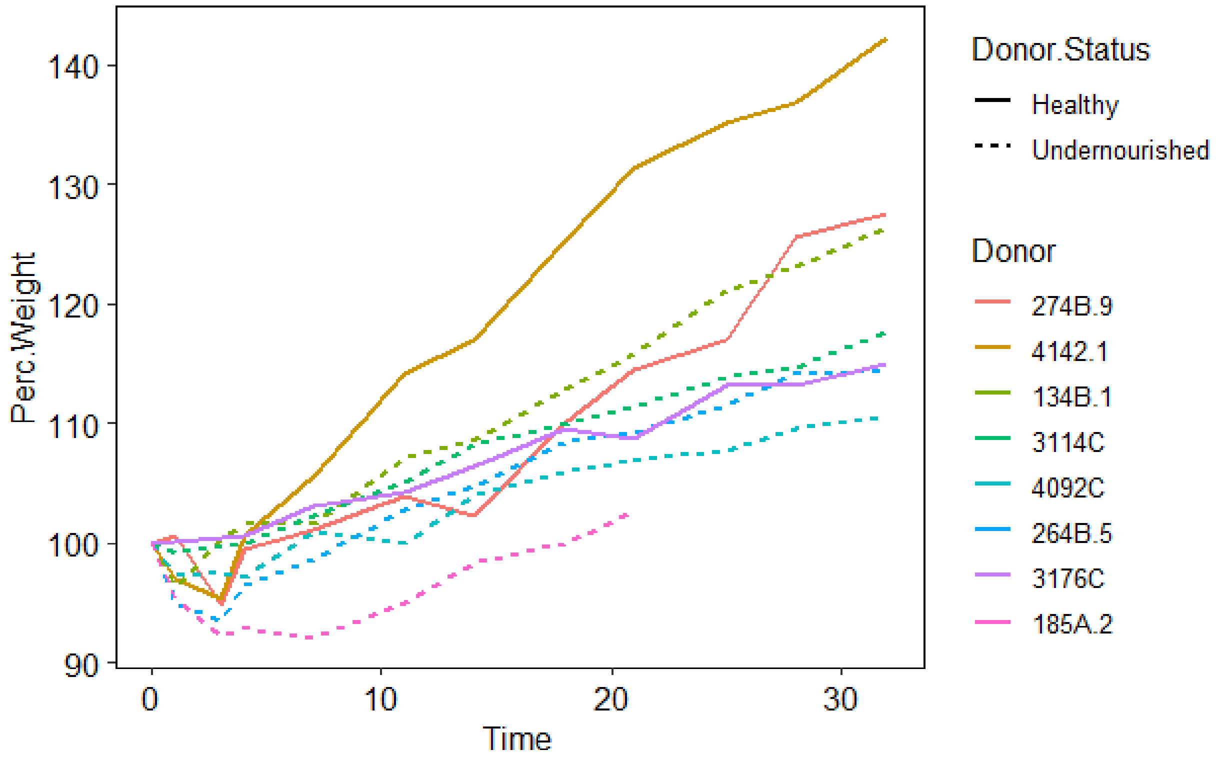

Fig. S4. EasyLME Exploratory Plots Figure 2. Average trends in the response variable (here Perc.Weight) over time colored by the higher-level random effect variable (here Donor) and differentiated by the grouping variable (here Donor.Status). This plot is helpful to visualize if a random intercept and/or random slope would be appropriate for the higher-level random effect variable. Missing data can also be identified by gaps in the lines or truncated lines. If a user's data is not nested, the plot will just show the trendlines for the random effect, without the need for averaging.

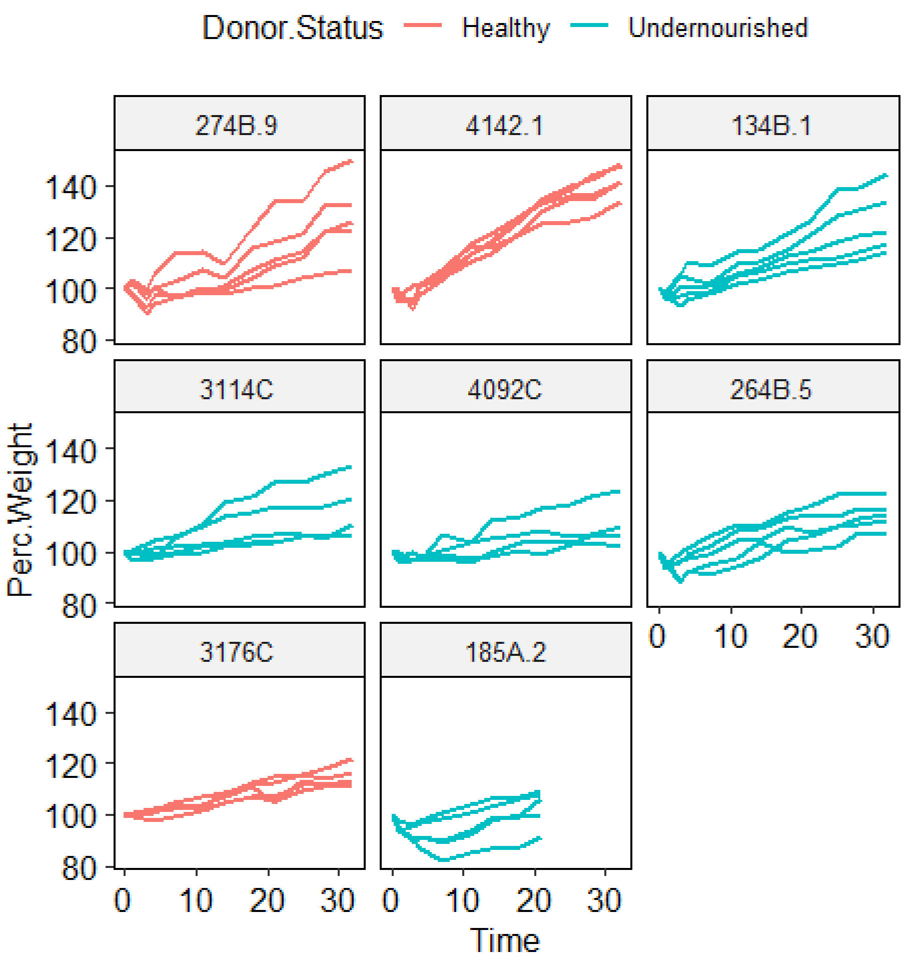

Fig. S5. EasyLME Exploratory Plots Figure 3. Trendlines in the response variable (here Perc.Weight) over time faceted by the higher-level random effect variable (here Donor) and colored by the grouping variable (here Donor.Status). These plots are helpful to visualize if a random intercept and/or random slope would be appropriate for the nested random effect variable (here Mouse). If a user's data is not nested, this plot will not be displayed.

Disease Models & Mechanisms • Supplementary information

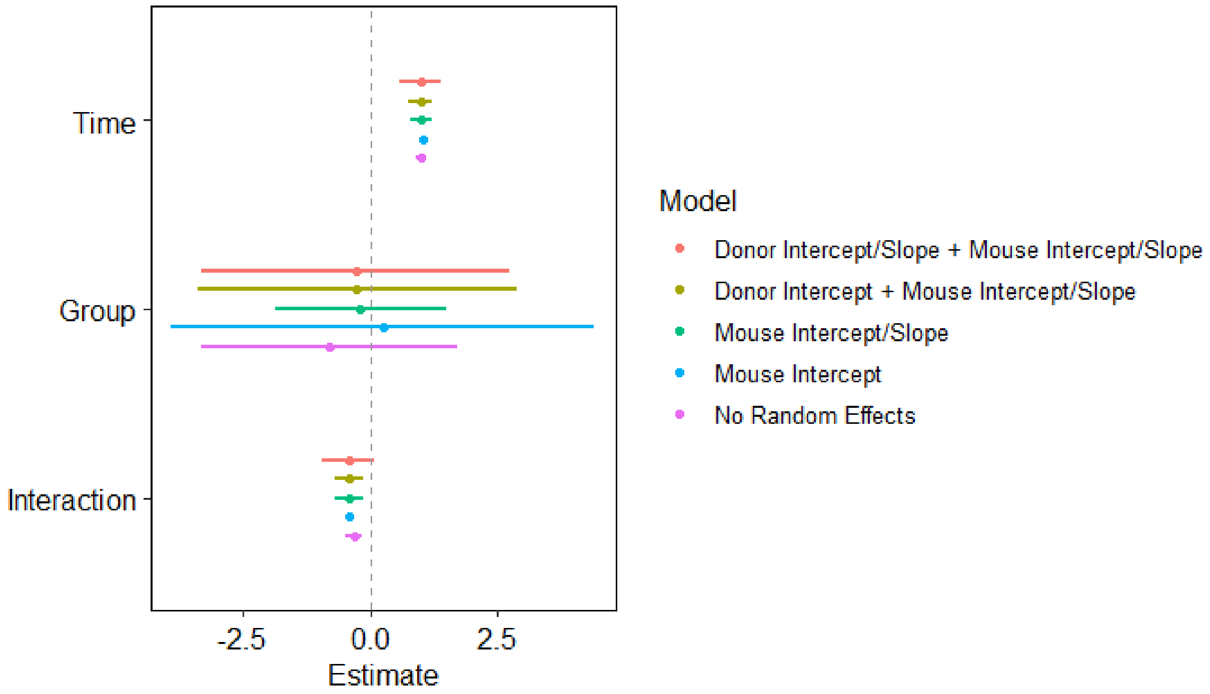

Fig. S6. EasyLME Model Results Figure 4. Coefficient plot of the fixed effect estimates in decreasing order of model complexity with 95% confidence intervals. This plot is helpful to visualize the information presented in the Model Results table.

Disease Models & Mechanisms • Supplementary information

Table S1. Type I error and power (N = 40) for the group by time interaction effect

	**Mouse Intercept & Slope Simulation Scenario**			**Mouse Intercept Simulation Scenario**			**No Random Effects Simulation Scenario**		
**Effect Size**	Mouse Intercept & Slope (Model III)	Mouse Intercept (Model IV)	No Random Effects (Model V)	Mouse Intercept & Slope (Model III)	Mouse Intercept (Model IV)	No Random Effects (Model V)	Mouse Intercept & Slope (Model III)	Mouse Intercept (Model IV)	No Random Effects (Model V)
0%*	0.047 [0.043, 0.051]	0.499 [0.490, 0.509]	0.314 [0.305, 0.323]	0.039 [0.035, 0.043]	0.051 [0.047, 0.055]	0.017 [0.014, 0.019]	0.038 [0.034, 0.042]	0.050 [0.046, 0.055]	0.049 [0.045, 0.054]
10%	0.06 [0.05, 0.08]	-**	-	0.36 [0.33, 0.39]	0.41 [0.38, 0.44]	0.24 [0.22, 0.27]	0.37 [0.34, 0.40]	0.40 [0.37, 0.43]	0.40 [0.37, 0.43]
25%	0.12 [0.10, 0.14]	-	-	0.99 [0.98, 0.99]	0.99 [0.98, 1.00]	0.97 [0.96, 0.98]	0.98 [0.97, 0.99]	0.99 [0.98, 0.99]	0.99 [0.98, 0.99]
50%	0.34 [0.31, 0.37]	-	-	1.00 [1.00, 1.00]	1.00 [1.00, 1.00]	1.00 [1.00, 1.00]	1.00 [1.00, 1.00]	1.00 [1.00, 1.00]	1.00 [1.00, 1.00]
100%	0.85 [0.83, 0.88]	-	-	1.00 [1.00, 1.00]	1.00 [1.00, 1.00]	1.00 [1.00, 1.00]	1.00 [1.00, 1.00]	1.00 [1.00, 1.00]	1.00 [1.00, 1.00]

*Type I Error
**Power is only compared when Type I Error was close to or below the expected level of 0.05.
